# Reliability analysis of subsea control system using FMEA and FFTA

**DOI:** 10.1038/s41598-023-42030-3

**Published:** 2024-12-02

**Authors:** Chao Liu, Guangxin Li, Wensheng Xiao, Jian Liu, Liping Tan, Changjiang Li, Teng Wang, Fengran Yang, Chengzhi Xue

**Affiliations:** 1 College of Electromechanical Engineering, Qingdao University of Science & Technology, Qingdao, 266061 China; 2 College of Mechanical and Electrical Engineering, China University of Petroleum (East China), Qingdao, 266580 China; 3 National Engineering Lab of Offshore Geophysical & Exploration Equipment, China University of Petroleum (East China), Qingdao, 266580 China; 4 Xuzhou Construction Machinery Group, Xuzhou, 221004 China

**Keywords:** Ocean sciences, Engineering

## Abstract

Reliability technology plays a significant role in ensuring the safe operation of the subsea control system. To perform a comprehensive analysis of the reliability of complex systems, a combination of Failure Mode and Effects Analysis (FMEA) and Fuzzy Fault Tree Approach (FFTA) is introduced. Firstly, the FMEA method is used to analyze the potential failure modes and causes of system failure by completing the qualitative analysis of system reliability from the perspective of multi-factor failure modes. And the risk matrix diagram is applied to determine the degree of harm of different failure modes to the system. Then, the system reliability is quantitatively analyzed using FFTA, and a fault tree model is established by dividing the system into "system-subsystem-component" and solving for the minimum cut set. In addition, the failure probability of the top-level event is quantitatively calculated by introducing fuzzy set theory, and the probabilistic importance of the bottom-level event is analyzed to find out the weak points of each subsystem. Finally, a qualitative and quantitative reliability analysis is conducted by using FMEA-FFTA method for subsea control system. Effective measures should be taken to focus on preventive protection and regular testing for the high risk, medium–high risk and medium risk modes for subsea control system.

## Introduction

With the continuous depletion of oil and gas resources, the major oil companies are turning to the development of offshore oil and gas resources, and the demand for offshore oil exploit equipment is rapidly rising^1,2^. As the mainstream mode of offshore oil and gas development, subsea oil and gas production systems are a series of equipment installed on the seabed to carry out oil and gas extraction operations, which have many advantages such as saving extraction costs, and improving oil and gas extraction efficiency^3^. As an important equipment for oil and gas development, the subsea control system is located in complex working environment, which not only be affected by multiple factors such as low temperature, high pressure and corrosion, but also be subject to risks such as falling objects from the sea and impacts from fishing nets, leading to failure modes such as leakage and corrosion^4,5^.

According to the statistics of major accidents over the years, an explosion of an offshore oil and gas system in the UK killed 167 people and caused economic losses of up to US$3.4 billion in 1988^6^. In 2005, a fire on an offshore drilling rig in India killed more than 10 people and caused a large amount of crude oil to leak, resulting in an oil pollution area of at least 15,000 square miles, with many rare marine species on the verge of extinction and considerable economic losses^7^. Major crude oil spills and explosions in the Gulf of Mexico in 2010 and 2016 caused economic losses of up to US$40 billion, resulting in irreversible pollution of the sea and ecological environment^8,9^. Failures in marine oil and gas production systems, such as leaks and explosions, have the potential to inflict significant economic losses and ecological harm, resulting in substantial adverse societal ramifications. Numerous incidents have already resulted in severe economic losses and environmental degradation, thereby amplifying concerns surrounding the safety and reliability of the equipment^10^. Therefore, it is necessary to carry out reliability analysis research of subsea control systems, further to provide effective measures and reliability technology guidance to prevent the occurrence of maritime accidents.

Reliability theory refers to an analytical method for assessing whether a system achieves a specified reliability over a specified period time and under specified conditions^11,12^. At the same time, it provides a way to measure the relative reliability of a system, and thus to identify weaknesses in that system and provide a theoretical basis for improving the design of system^13^. Reliability theory provides a way to predict system reliability from the component level to the system level^14,15^. System reliability models can be divided into combinatorial, state space and hierarchical models^16,17^. Complex systems are more demanding in conducting reliability analysis and assessment compared to other systems, and it is bound to be multi-level and multi-faceted^18,19^. In the twenty-first century, reliability research tends to be more integrated, systematic, collaborative and precise, conducting reliability assessment of complex systems has become a prominent issue.

In the field of engineering, there are currently three main methods of system reliability analysis: qualitative risk analysis, quantitative risk analysis and semi-qualitative risk analysis^20,21^. Qualitative risk analysis is based on the subjective experience of the researcher, and expert opinion is used to give the probability of failure of the event and the influencing factors, such as Hazard Checklist Method, Hazard and Operability Analysis Method, Pre-hazard Analysis Method, Hazard and Operability (HAZOP) Analysis, Failure Mode and Effect Analysis (FMEA)^22,23^. Quantitative risk analysis is mainly based on the reliability model established by the system failure statistics, and the reliability index is calculated by the reliability model, such as FTA method^24,25^, Reliability Block Diagrams (RBD)^26^, Markov Analysis^27^, Bayesian Networks^28^, Monte Carlo Simulation^29^, GO method^30^, Petri Nets^31^, the potential failure mode and effect criticality analysis (FMECA)^32^. Semi-quantitative risk analysis is a method between qualitative and quantitative analysis, such ETA (Event Tree Analysis) method^33^, Facility Risk View (FRR)^34^.

System reliability analysis methods can be categorized into several different approaches, each with its own strengths and limitations. A comparative study of these methods helps in understanding their applicability and effectiveness in different scenarios. Qualitative risk analysis is a typical fuzzy analysis method that enables a quick risk analysis of the system's hazards and a subjective ranking of the risks from a subjective perspective. Quantitative risk analysis is a quantification of the risk problem, with more specific and clear analysis objectives and a higher degree of accuracy. Semi-quantitative risk analysis uses a quantitative risk analysis, however, quantitative results are not available. In contrast, quantitative analysis is more rigorous and requires a wealth of mathematical theory and a sufficiently detailed database as a research tool. From the perspective of qualitative analysis, complex systems are characterized by multi-temporality, non-determinism, openness and chaos; from the perspective of quantitative analysis, complex systems are characterized by high order and high dimensionality.

Scholars have conducted researches in the design and analysis of subsea production system reliability. FMC, Cameron and other foreign companies have a lot of practical experience, statistical data and system reliability assessment methods in the subsea oil and gas systems. The American Petroleum Institute (API) gave a recommended practice for technical and risk management of subsea control system reliability-API RP 17N, which is a summary of international experience and research results on reliability and technical risk management of subsea control system. FMEA is a systematic approach used to identify the potential failure modes of each functional block of the system, and to study the impact of these failures on the system, which also provides valuable insights into potential failure modes and their impact, allowing for early mitigation and design improvements. Kolios et al. used FMEA to analyze the reliability of subsea control module (SCM), and reveal the key failure modes of SCM^35^. Singh et al. applied FMEA to study the fault modes, causes and effects of distribution transformers, and determined the risk priority of the most critical parts of distribution transformers^36^. Wanvik et al. used reliability analysis methods to calculate the lifetime and availability of the subsea production system^37^. Umofia proposed a multi-criteria approach to improve FMEA for the SCM^38^. FTA is a system failure-oriented analysis method, which can effectively analyze the causes of system failure and make qualitative and quantitative analysis of reliability and safety. Lavasani et al. proposed an improved FTA method for subsea pipelines to identify the underlying causes of undesired events and determine the logical relationships between the causes^39^. Hu et al. proposed a numerical approximation model based on FTA for oil and gas leakage risk analysis of subsea production systems^40^.

For other reliability methodology researches, Innal et al. used a segment-by-segment martensite process for dynamic risk assessment of subsea oil and gas production system^41^. Shukla et al. assessed the risks of offshore oil field development and production from a health, safety and environment (HSE) perspective^42^. Deyab et al. proposed a risk assessment method for offshore oil and gas extraction equipment operating in a harsh environment for a long period of time, which used Bayesian networks to address inter-event uncertainty^43^. Cai et al. performed a quantitative risk assessment based on Bayesian networks for subsea blowout preventer operations to obtain posterior probabilities^44^. Zuo et al. conducted a reliability study on parameter uncertainty in time-varying failure rates of subsea emergency shutdown systems^45^. Bhardwaj et al. proposed a Bayesian framework for predicting the reliability of subsea processing systems considering uncertain influencing factors^46^. Pang et al. developed a dynamic Bayesian network-based approach to assess the reliability and safety of subsea Christmas trees^47^. Guo et al. presented an agent-based dynamic reliability modeling method for subsea Christmas trees, considering fault propagation^48^. Wang et al. employed dynamic Bayesian networks and Monte Carlo simulation to evaluate the reliability of subsea wellhead connectors throughout their service life^49^. Wu et al. performed reliability analysis of subsea wellhead systems considering fatigue and degradation during their operational lifespan^50^. Srivastav et al. introduced degradation modeling in the qualification of novel subsea technologies^51^. Kong et al. developed a fault diagnosis methodology for redundant closed-loop feedback control systems, focusing on the subsea blowout preventer system^52^. Wang et al. presented an all-electric gate valve actuator for subsea production control systems, including the prototype development and testing^53^. Tao et al. proposed a fault diagnostic method for subsea control systems based on a digital twin approach, aiming to enhance system performance and reliability^54^.

Though scholars have conducted reliability analysis on subsea control system, most of them only consider the impact of some typical failure modes on the system, and the reliability analysis methods involved are relatively single. The traditional reliability analysis methods can only perform some qualitative analysis, and the applied traditional FTA method generally assumes that the event failure probability is an exact value, the calculation results have a large deviation from the actual situation. In general, the reliability analysis of subsea control systems is not sufficiently well established. As an essential part of the subsea production system, the subsea control system plays a critical role in ensuring the normal exploitation of subsea oil and gas, and it is vital for the safe of the subsea production system. Therefore, a composite reliability analysis method combining FMEA and Fuzzy Fault Tree Analysis (FFTA) is introduced to analyze the reliability of subsea control system in this study, which also offers several advantages for analyzing the reliability of complex systems:

1. Complementary analysis: FMEA-FFTA complement each other by addressing different aspects of system reliability. FMEA focuses on failure modes and their causes, while FFTA focuses on aggregating these failure modes to assess system-level reliability. By integrating the two methods, a more comprehensive understanding of system reliability can be achieved.
2. Enhanced risk assessment: FMEA-FFTA provides a more accurate and comprehensive assessment of system risks. FMEA helps in identifying critical failure modes and their effects, while FFTA quantifies the probability of system failure. This allows for a more reliable estimation of system risks and assists in making informed decisions regarding risk mitigation strategies.
3. Improved design optimization: By integrating FMEA and FFTA, potential design weaknesses can be identified early on and appropriate measures can be taken to improve system reliability.
4. Efficient resource allocation: By prioritizing critical failure modes identified through FMEA and FFTA, resources can be allocated more efficiently to mitigate high-risk failure modes, ensuring effective utilization of available resources.

In conclusion, the combination of FMEA and FFTA provides a powerful framework for analyzing the reliability of complex systems. It offers a comprehensive understanding of failure modes, their causes, and their quantifiable impact on system-level reliability. This integrated approach facilitates better decision-making, efficient resource allocation, and improved system design optimization.

Then, the FMEA-FFTA is carried out systematically in terms of failure modes, failure causes and failure components, and the most authoritative reliability database Offshore Reliability Data (OREDA) is consulted to analyze the severity of subsea control system failure modes and identify system weaknesses in a qualitative and quantitative way. The analysis will provide some reference significance for the reliability design of subsea control system.

The rest sections of this study are arranged as follows. Section "Subsea control systems" introduces the overview of subsea control systems. Section "Failure analysis using FMEA and FFTA" shows failure analysis using FMEA and FFTA. Section "Reliability analysis of subsea control system" conducts a reliability analysis of the subsea control system. Section "Conclusion" introduces conclusion.

## Subsea control systems

### Subsea oil and gas production system

**Figure 1:**
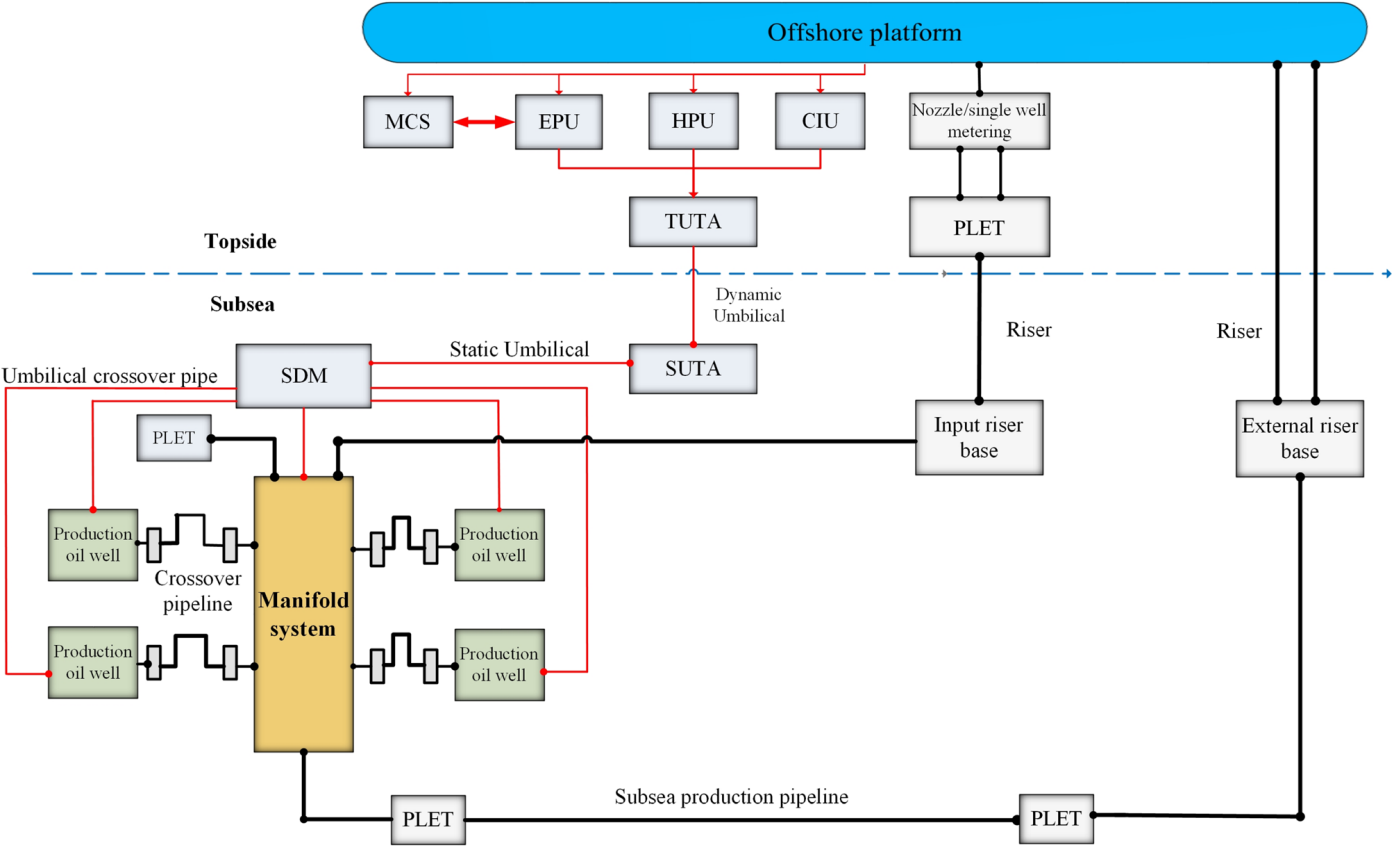
A typical model of the subsea oil and gas production system.

The subsea oil and gas production system refers to a series of equipment installed on the seabed and carrying out oil recovery operation, it can be divided into five major subsystems: the subsea control system, the riser system, the subsea pipeline system, the subsea pipe manifold system, the subsea wellhead device and the Christmas tree system. A typical subsea oil and gas production system model based on the functions and interactions between the different systems, as shown in Fig. 1.

In Fig. 1, the red line segments represent the line network of the subsea control system and the chemical injection lines. The black lines represent the oil, gas and water transport lines and the water injection lines. The subsystems interact with each other through terminal interfaces to transport the extracted oil, gas and water multiphase fluids from the seabed to the offshore platform for processing.

### Subsea control system

The subsea control system consists of three major parts: surface control module, subsea control module and control umbilical, in which the surface control module mainly includes hydraulic power unit (HPU), electric power/signal unit (EPU), Chemical injection unit, and main control station (MCS). The subsea control module includes subsea distribution unit (SDU), subsea control module (SCM), and subsea sensors, etc. The control umbilical section mainly consists of the topside umbilical termination unit (TUTA), subsea umbilical termination unit (SUTA), the static umbilical and dynamic umbilical. The operation principle of the subsea control system is shown as fellow.

1. Signal transmission: control signals are sent from the MCS of the offshore platform, coded and transmitted to the SCM by umbilical, which is decoded and then executed.
2. Hydraulic transmission: hydraulic fluid is transmitted down through the HPU via the umbilical to the SDU, which is distributed and delivered to each SCM to enable the hydraulic pressure to achieve remote control operations.
3. Chemical transmission: chemical is injected from the SUTA and transmitted to the SDU, then distributed at the terminal according to the needs of the subsea production equipment.

**Figure 2:**
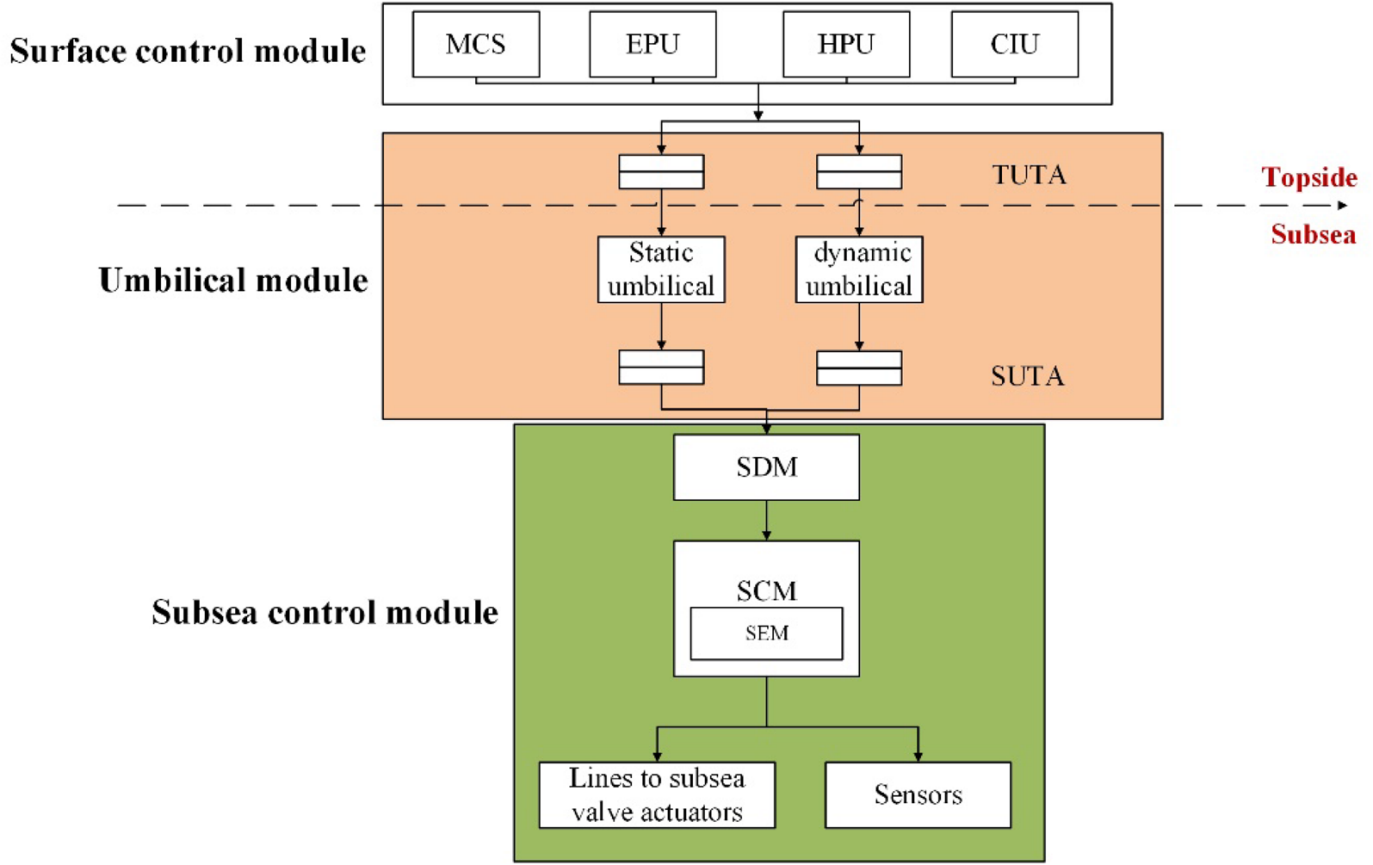
Schematic diagram of subsea control system.

Based on the operation principle and function of the subsea control system, a block diagram of its functional structure is constructed, as shown in Fig. 2.

#### Surface control module

**Figure 3:**
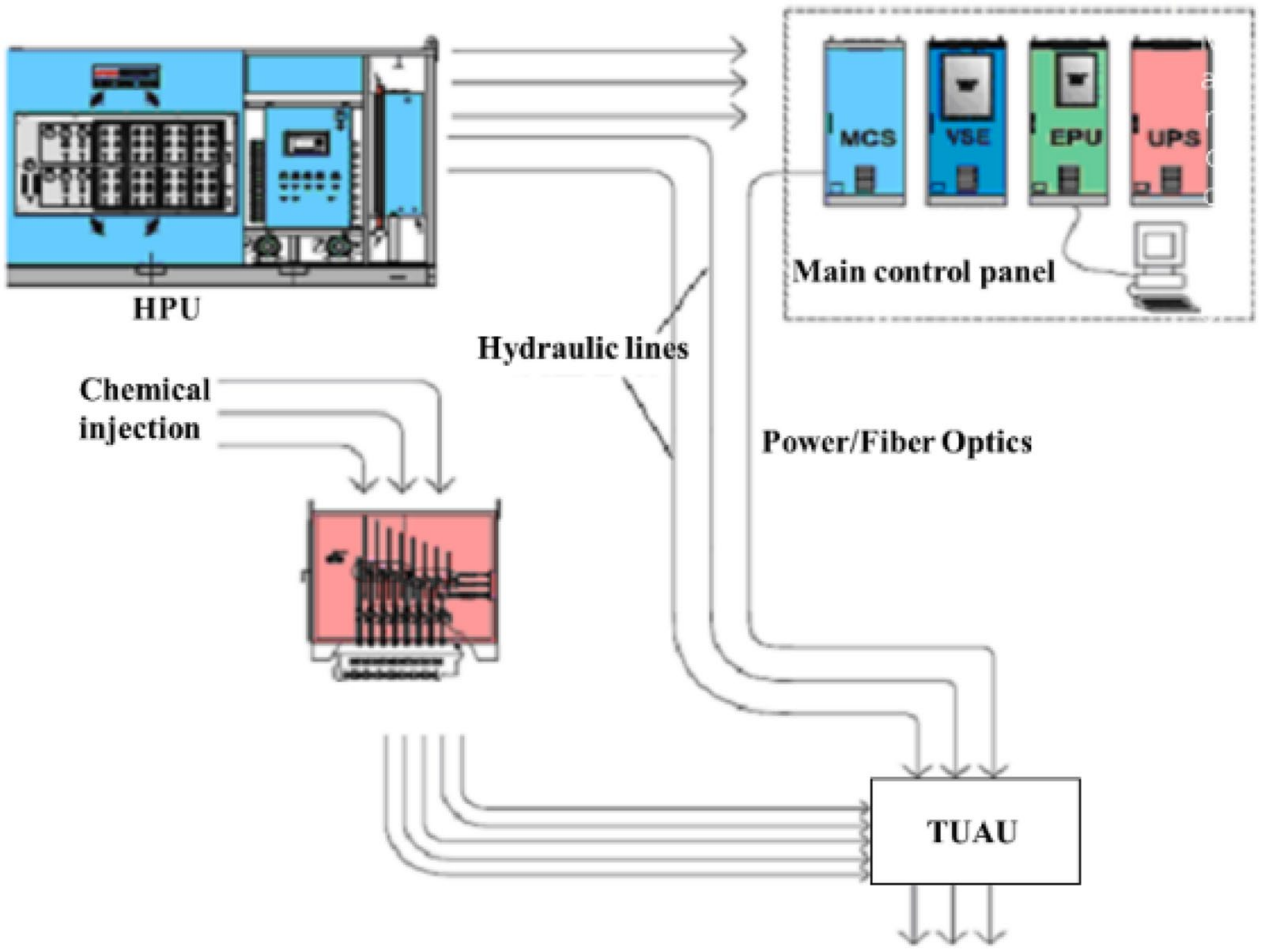
Surface control module.

The surface control module mainly includes: main control station (MCS), hydraulic power unit (HPU), electric power/signal unit (EPU), and Chemical injection unit (CIU), as shown in Fig. 3.

1. MCS: MCS usually consists of the computer, the display and the control section. it provides the interaction between the operator and the subsea equipment. The operator can control the MCS through a human–machine interface consisting of a keyboard and visualization operations.
2. EPU: EPU generally consists of ventilation unit, transmission monitoring and power isolation unit, controller unit, power unit, etc., which is equipped with control, electrical isolation and detection functions. EPU provides online detection and alarm of input and output voltage and current, insulation monitoring and alarm and trip protection, it also provides redundant, independent and single-phase electrical power to the subsea production system.
3. HPU: HPU contains the supply tank, return tank, low pressure hydraulic control system, high pressure oil pump system, hydraulic oil cabinet, accumulator and circulation pumps for oil filling and flushing. HPU is primarily used to provide a stable, clean supply of pressure hydraulic fluid to the subsea production facility via the umbilical.
4. CIU: Subsea chemicals are injected from the subsea umbilical terminals into the subsea distribution system, which dispenses chemicals to each well or manifold terminal, it also supplies and discharges fluids for pressure testing and pressure balancing of flow control equipment.

#### Subsea control module

1. SCM: SCM generally adopts a modular design and is a highly integrated technical product combining mechanics, electricity and hydraulics. It generally consists of an upper top plate, lifting mechanism, protection cylinder, internal support parts, electronic device module (SEM), pressure compensator, hydraulically integrated valve block, mounting base plate and locking mechanism. SCM is used to control the subsea production equipment such as subsea oil trees, subsea pipe manifolds and subsea separators by executing the commands from the MCS.
2. SDU: SDU is the essential equipment for the subsea control system and consists of the electrical distribution module, the hydraulic distribution module, the umbilical termination joint and the lower foundation, which is connected upstream to the umbilical and downstream to the oil production tree.
3. Subsea sensors: Subsea sensors mainly include pressure and temperature sensor, flow sensor, pressure sensor, sand detection sensor, temperature sensor, valve position sensor, etc.

#### Umbilical module

Umbilical module mainly consists of the TUTA, SUTA, the static umbilical and the dynamic umbilical. Umbilical module is constructed as an external negative protection structure with an electrical connector, crossover tube, hydraulic fluid distribution valve and chemical injection dispenser, operated via an ROV operator panel. The umbilical is the 'lifeline' between the topside facility and the subsea production system and can be divided into static umbilical and dynamic umbilical.

The main roles of umbilical include providing hydraulic power channels for subsea valve actuators, generating electrical power for control boxes, electric pumps etc., providing remote control and monitoring data transmission channels for subsea facilities and wells. Static umbilical requires consideration of seabed hydrodynamic stability, fallout and stranding effects, environmental loads, torsional balance, etc. Dynamic umbilical requires consideration of fatigue strength due to eddy vibration.

## Failure analysis using FMEA and FFTA

### FMEA

Failure Mode and Effects Analysis (FMEA) is a bottom-up reliability qualitative analysis method based on a pre-agreed minimum level, mainly used for multi-factor failure mode analysis of systems. FMEA is an inductive analysis method that identifies the causes of each failure, and classifies each failure mode according to its hazard level, ease of detection and frequency of occurrence^55,56^. A risk matrix is used to determine the degree of harm to the system's functionality of the different failure modes and to identify the level of risk of failure.

1. Preliminary stage work: The main task is to carry out preparatory work, such as collecting relevant information about the analysis object, constructing the functional structure block diagram of each subsystem, and formulating the overall FMEA analysis plan.
2. Medium-term work: To construct failure factors and failure data statistics for the study population, analyze all possible lowest level failure modes and causes of failure, identify all potential failure modes in the system and investigate the causes of failure.
3. Post stage work: The failure severity evaluation criteria and frequency evaluation criteria are established, and the risk matrix method is used to rank the failure modes by risk. Then, reasonable improvement and refinement measures are proposed for each failure mode, and finally the FMEA table is output.

**Figure 4:**
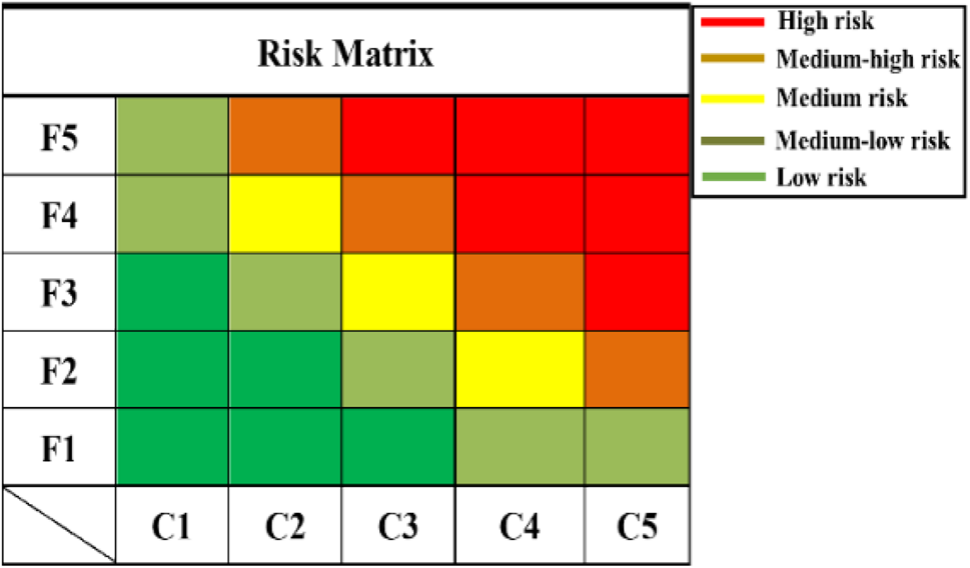
Risk matrix table.

The risk matrix method is used to comprehensively identify failure modes, and the failure severity evaluation criteria, occurrence evaluation criteria and risk matrix tables are first developed prior to the system analysis, as shown in Tables 1 and 2. A multi-factor failure mode analysis is carried out on the system in accordance with the FMEA analysis. The risk matrix method is used to comprehensively identify the hazard level of the failure mode and classify the failure mode into five levels: High risk, Medium–high risk, Medium risk, Medium–low risk and Low risk, as shown in Fig. 4.

**Table 1:** Failure degree evaluation criteria.

Level	Type	Classification criteria
F5	Frequent happen	The probability of a particular failure mode happening is greater than 20% of the total system failure probability
F4	Sometimes happen	The probability of a particular failure mode occurring is greater than 10% and less than 20% of the total system failure probability
F3	Occasionally happen	The probability of a particular failure mode happening is greater than 1% and less than 10% of the total system failure probability
F2	Less frequent happen	The probability of a particular failure mode occurring is greater than 0.1% and less than 1% of the total system failure probability
F1	Rarely happen	Less than 0.1% of the probability of a failure mode being greater than the total system failure probability

**Table 2:** Fault severity evaluation criteria.

Level	Type	Classification criteria
C5	Catastrophic failure	Major accidents occur in the system, even leading to aircraft destruction and serious environmental damage
C4	Fatal failure	It may cause considerable damage to systems and the environment, resulting in longer downtime, without posing a serious threat to life
C3	Serious failure	It may potentially lead to a reduction in system functionality, resulting in a smaller loss of overall system and environment
C2	Critical failure	It may potentially lead to degradation of system function with no apparent damage to the system, causing short downtime
C1	Mild failure	It may cause a slight degradation of the system's function, does not cause downtime and poses no danger to personnel

### FFTA

#### FTA

Fault Tree Analysis (FTA) is a graphical deductive logic reasoning method that uses a fault tree as a top-down decomposition of the system reliability analysis model^57,58^. By analyzing various factors that may lead to product failure in the product design process, the causes of product failure and its various possible combinations are identified. The probability of their occurrence is also quantified, and corresponding corrective measures are taken to improve the reliability of the system. FTA is a graphical deduction method, some basic symbols need to be defined before building the fault tree, generally including event symbols and logic gate symbols, event gate symbols and their meanings are shown in Table 3.

**Table 3:** Event symbols and significance.

Name	Symbols	Implication
Top event		The top event of the fault tree and the most unwanted event
Intermediate event		Events in the middle of the top and bottom events of the fault tree, both as input and output events
Bottom event		Also known as a basic event, it is at the bottom of the fault tree and is only used as an input event to a logic gate
Transfer symbol		To avoid repetitive diagrams, the fault tree structure is simplified using transfer symbols

FTA takes the least desired fault state of the system as the target of the logical analysis, called the top event. All possible direct causes are then identified, which are called intermediate events in the fault tree. The fault tree is traced back to all component states that caused the intermediate events to occur, which are called bottom events. The corresponding symbols and logics are used to connect the top event, middle event and bottom event into a tree logic diagram. The logical and causal relationships between events in a fault tree need to be represented by logic gates, the logic gate symbols and meanings of which are shown in Table 4.

**Table 4:** Logic gate symbol and significance.

Name	Symbols	Implication
And door		It means that input events X1 and X2 both occur before output event A occurs
Or door		It means that the output event A will occur when either of the input events X1, X2 occurs
Conditions and doors		It means that if the input events X1 and X2 both occur, the output event A will only occur if condition B is also satisfied
Condition or door		Under the condition that either of the input events X1 and X2 occurs, condition B must also be met for the output event A to occur

The general steps of the FTA method are described as follows.

1. Pre-preparation stage: Define the research object, familiarize with the system, and collect information related to system faults.
2. Middle tree construction stage: Determine the top event, middle event and bottom event of the fault tree, and construct a fault tree model of the system.
3. Post-analysis stage: Carry out qualitative and quantitative analysis of FTA, solve the minimum cut set, and calculate the probability of occurrence of the top event and the importance of the bottom event.

Before performing the system fault tree analysis, several assumptions need to be made: system and components have only a binary state of fault and normal and are represented by zeros and ones; failures between components are independent of each other. If ${\text{X}}_{1} ,{\text{X}}_{2} ,{\text{X}}_{3} \ldots {\text{X}}_{n}$ is used to represent the basic event in the fault tree, the top event state variable is represented by $\varphi (x)$, the bottom event state variable is represented by $x_{i}$, and the independent variable $x_{i}$ determines the top event state variable $\varphi (x)$, as shown in Eq. (1).

1 $$\varphi \left( x \right) = \varphi \left( {x_{1} ,x_{2},\ldots,x_{n} } \right)$$

Typical fault tree structure functions are "And" gates and "Or" gates, as shown in Eqs. (2) and (3)

2 $$\varphi \left( x \right) = \bigcap\limits_{i = 1}^{n} {x_{i} } ,\quad i = 1,2, \ldots ,n$$

3 $$\varphi (x) = \bigcup\limits_{i = 1}^{n} {x_{i} } ,\quad i = 1,2, \ldots ,n$$

Suppose that there are *n* bottom events ${\text{X}}_{1} ,{\text{X}}_{2} ,{\text{X}}_{3} \ldots {\text{X}}_{n}$ in the fault tree, and ${\text{C}} \in \left\{ {{\text{X}}_{1} ,{\text{X}}_{2} ,{\text{X}}_{3} \ldots {\text{X}}_{m} } \right\}$ is the set of partial bottom events. If T no longer occurs when any event ${\text{X}}_{{\text{i}}}$ is removed from the cut set C, this is said to be the minimum cut set. The qualitative analysis of fault trees focuses on finding the minimum cut set to analyze the weaknesses of the system. The minimum road set is the opposite of the minimum cut set, it is the minimum set of bottom events that make the top event T not occur and is mainly used to find the optimal and reasonable solution for the system.

#### Fuzzy FTA

The basic idea of fuzzy set theory is to fuzzily the absolute affiliation of an element to a set as contained in classical set theory^59^. The degree of subordination of an element $x$ to a set A is not just 0 or 1, rather it is any value in the interval 0 to 1, as shown in Eq. (4).

4 $$\mu_{{\text{A}}} = \left\{ \begin{array}{*{20}ll} 1&{\text{if}}\ x \in {\text{A}} \\ 0 < \mu_{{\text{A}}} \left( x \right) < 1&{\text{if }}\ x{\text{ belongs to a certain degree to A}} \\ 0& \text{if } x \notin {\text{A}}\end{array} \right.$$

in which, $\mu_{{\text{A}}}$ represents the strength of affiliation of $x$ to set A. L-R type fuzzy function is expressed as shown in Eq. (5) and is also denoted as $A = \left( {m,\alpha ,\beta } \right)_{LR}$^59^.

5 $$\mu_{{\text{A}}} \left( x \right) = \left\{ {\begin{array}{*{20}l} {{\text{L}}\left[ {\left( {m - x} \right)/\alpha } \right],x \le m,\alpha > 0} \\ {{\text{R}}\left[ {\left( {x - m} \right)/\beta } \right],x > m,\beta > 0} \\ \end{array} } \right.$$

in which, $\mu_{{\text{A}}} \left( x \right) \in \left[ {0,1} \right]$, *m* means the mean value of $A$, $\alpha ,\beta$ means the left and right edges of the fuzzy interval, then $\left( {\left. {m - \alpha } \right),\left( {m{ + }\beta } \right.} \right)$ means the upper and lower limits of the fuzzy interval. L-R type fuzzy functions are commonly normal, triangular and pointed, as shown in Eqs. (6)–(8).

Normal fuzzy function:

6 $$\left\{ {\begin{array}{*{20}l} {{\text{L}}\left[ {\left( {m - x} \right)/\alpha } \right] = \exp \left[ { - \left( {\left( {m - x} \right)/\alpha } \right)^{2} } \right],\quad x \le m,\alpha > 0} \\ {{\text{R}}\left[ {\left( {x - m} \right)/\beta } \right] = \exp \left[ { - \left( {\left( {m - x} \right)/\beta } \right)^{2} } \right],\quad x > m,\beta > 0} \\ \end{array} } \right.$$

Triangular fuzzy function:

7 $$\left\{ {\begin{array}{*{20}l} {{\text{L}}\left[ {\left( {m - x} \right)/\alpha } \right] = \max \left[ {0,1 - \left( {m - x} \right)/\alpha } \right],x \le m,\alpha > 0} \\ {{\text{R}}\left[ {\left( {x - m} \right)/\beta } \right] = \max \left[ {0,1 - \left( {m - x} \right)/\beta } \right],x > m,\beta > 0} \\ \end{array} } \right.$$

Pointed fuzzy function:

8 $$\left\{ {\begin{array}{*{20}l} {{\text{L}}\left[ {\left( {m - x} \right)/\alpha } \right] = {1 \mathord{\left/ {\vphantom {1 {\left[ {1{ + }\left( {m - x} \right)/\alpha } \right]}}} \right. \kern-0pt} {\left[ {1{ + }\left( {m - x} \right)/\alpha } \right]}},\quad x \le m,\alpha > 0} \\ {{\text{R}}\left[ {\left( {x - m} \right)/\beta } \right] = {1 \mathord{\left/ {\vphantom {1 {\left[ {1{ + }\left( {m - x} \right)/\beta } \right]}}} \right. \kern-0pt} {\left[ {1{ + }\left( {m - x} \right)/\beta } \right]}},\quad x > m,\beta > 0} \\ \end{array} } \right.$$

Set $\tilde{M} = \left( {m,\alpha ,\beta } \right)$, $\tilde{N} = \left( {n,\gamma ,\delta } \right)$ to be L-R fuzzy numbers and the algebraic algorithm is shown in Eqs. (9)–(12).

(1) Addition:

9 $$\tilde{M} \left( + \right)\tilde{N} = \left( {m,\alpha ,\beta } \right)\left( + \right)\left( {n,\gamma ,\delta } \right) = \left( {m + n,\alpha + \gamma ,\beta + \delta } \right)$$

(2) Subtraction:

10 $$\tilde{M} \left( - \right)\tilde{N} = \left( {m,\alpha ,\beta } \right)\left( - \right)\left( {n,\gamma ,\delta } \right) = \left( {m - n,\alpha - \gamma ,\beta - \delta } \right)$$

(3) Multiplication:

11 $$\tilde{M} \left( \times \right)\tilde{N} = \left( {m,\alpha ,\beta } \right)\left( \times \right)\left( {n,\gamma ,\delta } \right) \approx \left( {m \times n,m\gamma + n\alpha ,m\delta + n\beta } \right)$$

(4) Division:

12 $$\tilde{M} \left( \div \right)\tilde{N} = \left( {m,\alpha ,\beta } \right)\left( \div \right)\left( {n,\gamma ,\delta } \right) \approx \left( {\frac{m}{n},\frac{m\delta + n\alpha }{{n^{2} }},\frac{m\gamma + n\beta }{{n^{2} }}} \right)$$

Fuzzy fault tree analysis (FFTA) differs from traditional fault trees in that it expresses the bottom event failure probability as a fuzzy number and replaces the traditional logic gate operator with a fuzzy gate operator to solve for the top event failure probability, the fuzzy operator expression for the logic gate is shown in Eqs. (13)–(17).

(1) "And the door" fuzzy operator:

13 $$\begin{aligned} \mathop {F_{s}^{and} }\limits^{ \sim } = \prod\limits_{i = 1}^{n} {\mathop {F_{i} }\limits^{ \sim } } & = \left( {m_{1} ,\alpha_{1} ,\beta_{1} } \right)_{LR} \left( {m_{2} ,\alpha_{2} ,\beta_{2} } \right)_{LR} \cdot \cdot \cdot \left( {m_{n} ,\alpha_{n} ,\beta_{n} } \right)_{LR} \\ & = \left( {m_{{s_{i - 1} }} m_{i} ,m_{{s_{i - 1} }} \alpha_{i} + m_{i} \alpha_{{s_{i - 1} }} ,m_{{s_{i - 1} }} \beta_{i} + m_{i} \beta_{{s_{i - 1} }} } \right)_{LR} \\ & = \left( {m_{{s_{i} }} ,\alpha_{{s_{i} }} ,\beta_{{s_{i} }} } \right)_{LR} \\ \end{aligned}$$

in which, $m_{{s_{i} }} ,\alpha_{{s_{i} }} ,\beta_{{s_{i} }} \left( {i = 1,2,\ldots,n} \right)$ is expressed by Eq. (14).

14 $$\begin{aligned} m_{{S_{1} }} & = m_{1} ,m_{{S_{2} }} = m_{1} m_{2} ,m_{{S_{3} }} = m_{{S_{2} }} m_{3} ,, \cdot \cdot \cdot , \\ m_{{S_{i} }} & = m_{{S_{i - 1} }} m_{i} \\ \alpha_{{S_{1} }} & = \alpha_{1} ,\alpha_{{S_{2} }} = m_{1} \alpha_{2} + m_{2} \alpha_{1} ,\alpha_{{S_{3} }} = m_{{S_{2} }} \alpha_{3} + m_{2} \alpha_{{S_{2} }} , \cdot \cdot \cdot , \\ \alpha_{{S_{i} }} & = m_{{S_{i - 1} }} \alpha_{i} + m_{i} \alpha_{{S_{i - 1} }} \\ \beta_{{S_{1} }} & = \beta_{1} ,\beta_{{S_{2} }} = m_{1} \beta_{2} + m_{2} \beta_{1} ,\beta_{{S_{3} }} = m_{{S_{2} }} \beta_{3} + m_{2} \beta_{{S_{2} }} , \cdot \cdot \cdot , \\ \beta_{{S_{i} }} & = m_{{S_{i - 1} }} \beta_{i} + m_{i} \beta_{{S_{i - 1} }} \\ \end{aligned}$$

(2) "Or door" fuzzy operator:

15 $$\begin{aligned} \mathop {F_{s}^{or} }\limits^{ \sim } & = 1 - \prod\limits_{i = 1}^{n} {\left( {1 - \mathop {F_{i} }\limits^{ \sim } } \right)} = \left( {1,0,0} \right)_{LR} - \left\{ {\left[ {\left( {1,0,0} \right)_{LR} - \left( {m_{1} ,\alpha_{1} ,\beta_{1} } \right)_{LR} } \right]} \right\} \\ & \cdot \left[ {\left( {1,0,0} \right)_{LR} - \left( {m_{2} ,\alpha_{2} ,\beta_{2} } \right)_{LR} } \right] \cdot \cdots \cdot \left[ {\left( {1,0,0} \right)_{LR} - \left( {m_{n} ,\alpha_{n} ,\beta_{n} } \right)_{LR} } \right] \\ \end{aligned}$$

Or it can be simply written in recursive form as follows.

16 $$\begin{aligned} \mathop {F_{s}^{or} }\limits^{ \sim } & = \left( {m_{s} ,\alpha_{s} ,\beta_{s} } \right)_{LR} = \left( {1,0,0} \right)_{LR} - \left[ \begin{gathered} m_{{s_{i - 1} }} \left( {1 - m_{i} } \right),m_{{s_{i - 1} }} \alpha_{i} + \left( {1 - m_{i} } \right)\alpha_{{s_{i - 1} }} \hfill \\ ,m_{{s_{i - 1} }} \beta_{i} + \left( {1 - m_{i} } \right)\beta_{{s_{i - 1} }} \hfill \\ \end{gathered} \right]_{LR} \\ & = \left( {1,0,0} \right)_{LR} - \left( {m_{{s_{i} }} ,\alpha_{{s_{i} }} ,\beta_{{s_{i} }} } \right)_{LR} \\ \end{aligned}$$

in which,$m_{{s_{i} }} ,\alpha_{{s_{i} }} ,\beta_{{s_{i} }}$ is expressed by Eq. (17).

17 $$\begin{aligned} m_{{S_{1} }} & = m_{1} ,m_{{S_{2} }} = \left( {1 - m_{1} } \right)\left( {1 - m_{2} } \right), \\ m_{{S_{3} }} & = m_{{S_{2} }} \left( {1 - m_{3} } \right),, \cdot \cdot \cdot ,m_{{S_{i} }} = m_{{S_{i - 1} }} \left( {1 - m_{i} } \right) \\ \alpha_{{S_{1} }} & = \alpha_{1} ,\alpha_{{S_{2} }} = \left( {1 - m_{1} } \right)\alpha_{2} + \left( {1 - m_{2} } \right)\alpha_{1} ,\alpha_{{S_{3} }} = m_{{S_{2} }} \alpha_{3} + \left( {1 - m_{3} } \right)\alpha_{{S_{2} }} , \cdot \cdot \cdot , \\ \alpha_{{S_{i} }} & = m_{{S_{i - 1} }} \alpha_{i} + \left( {1 - m_{i} } \right)\alpha_{{S_{i - 1} }} \\ \beta_{{S_{1} }} & = \beta_{1} ,\beta_{{S_{2} }} = \left( {1 - m_{1} } \right)\beta_{2} + \left( {1 - m_{2} } \right)\beta_{1} , \\ \beta_{{S_{3} }} & = m_{{S_{2} }} \beta_{3} + \left( {1 - m_{3} } \right)\beta_{{S_{2} }} , \cdot \cdot \cdot ,\beta_{{S_{i} }} = m_{{S_{i - 1} }} \beta_{i} + \left( {1 - m_{i} } \right)\beta_{{S_{i - 1} }} \\ \end{aligned}$$

**Figure 5:**
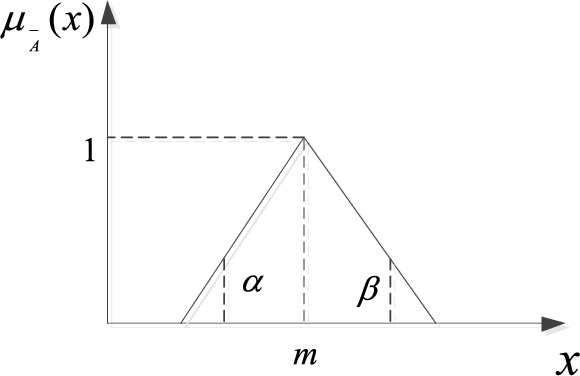
Schematic diagram of the triangular affiliation function.

The triangular fuzzy function has the advantages of easy expression and simple operation. Therefore, the triangular fuzzy number is used to describe the probability of fault events in this study, which can effectively avoid the shortcomings of the traditional analysis method of the existence of fault probability data and inaccuracy^59^, as shown in Eqs. (18), (19), and Fig. 5.

18 $$\left\{ {\begin{array}{*{20}l} {{\text{L}}\left[ {\left( {m - x} \right)/\alpha } \right] = \max \left[ {0,1 - \left( {m - x} \right)/\alpha } \right],\quad x \le m,\alpha > 0} \\ {{\text{R}}\left[ {\left( {x - m} \right)/\beta } \right] = \max \left[ {0,1 - \left( {m - x} \right)/\beta } \right],\quad x > m,\beta > 0} \\ \end{array} } \right.$$

19 $$\mu_{A} \left( x \right) = \left\{ {\begin{array}{*{20}ll}0&\quad x < m - \alpha \hfill \\ 1 - \left( {m - x} \right)/\alpha ,&\quad m - \alpha \le x \le m \hfill \\ 1 - \left( {x - m} \right)/\beta ,&\quad m < x \le m + \beta \hfill \\ 0,&\quad x > m + \beta \hfill \\ \end{array} } \right.$$

Assuming that $A_{\lambda } = \left\{ {u\left| {u \in U,A\left( u \right) \ge \lambda } \right.} \right\}$, then called $A_{\lambda }$ a $\lambda$-intercept set of A^59^. $\lambda$ is called the confidence level, as shown in Eq. (20).

20 $$A_{\lambda } = \left[ {\left( {m - \alpha ) + \alpha \cdot \lambda ,(m + \beta } \right) - \beta \cdot \lambda } \right]$$

The intercept set of the bottom event failure probability $F_{i}$ is shown in Eq. (21).

21 $$\begin{gathered} F_{i} = \left[ {\left( {m_{1} - \alpha_{1} } \right) + \alpha_{1} \lambda ,\left( {m_{1} + \beta_{1} } \right) - \beta_{1} \lambda } \right] \hfill \\ \begin{array}{*{20}c} {\begin{array}{*{20}c} {\begin{array}{*{20}c} {} & {} & {} & {} \\ \end{array} } & {} & {} & { \cdot \cdot \cdot } \\ \end{array} } & {} & {} & {} \\ \end{array} \hfill \\ F_{n} = \left[ {\left( {m_{n} - \alpha_{n} } \right) + \alpha_{n} \lambda ,\left( {m_{n} + \beta_{n} } \right) - \beta_{n} \lambda } \right] \hfill \\ \end{gathered}$$

In FTA, the logical gate fuzzy operator for the bottom event containing the probability of a $\lambda$ truncated set of faults is calculated as shown in Eqs. (22)–(23).

(1) " And door " structure:

22 $$\begin{aligned} F_{s}^{and} & = \prod\limits_{i = 1}^{n} {\left( {F_{i} } \right)} \\ & = \prod\limits_{i = 1}^{n} {\left[ {\left( {m_{i} - \alpha_{i} } \right) + \alpha_{i} \lambda ,\left( {m_{i} + \beta_{i} } \right) - \beta_{i} \lambda } \right]} \\ & = \left[ {\prod\limits_{i = 1}^{n} {\left[ {\left( {m_{i} - \alpha_{i} } \right) + \alpha_{i} \lambda } \right]} ,\prod\limits_{i = 1}^{n} {\left[ {\left( {m_{i} + \beta_{i} } \right) - \beta_{i} \lambda } \right]} } \right] \\ \end{aligned}$$

(2) " Or door " structure:

23 $$\begin{aligned} F_{s}^{or} & = 1 - \prod\limits_{i = 1}^{n} {\left( {1 - F_{i} } \right)} \\ & = \left[ {1,1} \right] - \prod\limits_{i = 1}^{n} {\left\{ {\left[ {1,1} \right] - \left[ {\left( {m_{i} - \alpha_{i} } \right) + \alpha_{i} \lambda ,\left( {m_{i} + \beta_{i} } \right) - \beta_{i} \lambda } \right]} \right\}} \\ & { = }\left[ {1 - \prod\limits_{i = 1}^{n} {\left[ {1 - \left( {m_{i} - \alpha_{i} } \right) - \alpha_{i} \lambda } \right]} ,1 - \prod\limits_{i = 1}^{n} {\left[ {1 - \left( {m_{i} + \beta_{i} } \right) + \beta_{i} \lambda } \right]} } \right] \\ \end{aligned}$$

According to the basic theory of reliability, the functional relationship between reliability $R\left( t \right)$ and failure rate $\lambda \left( t \right)$ can be expressed as shown in Eq. (24)^59^.

24 $$R\left( t \right) = e^{{ - \int_{0}^{\infty } {\lambda \left( t \right)dt} }} = \exp \left( { - \int_{0}^{\infty } {\lambda \left( t \right)dt} } \right)$$

Probabilistic importance is the trend of the bottom event failure rate relative to the top event failure rate, reflecting the importance of the bottom event relative to the top event in the fault tree, as shown in Eq. (25).

25 $$I_{h} \left( j \right) = \frac{\partial h\left( p \right)}{{\partial p_{i} }},\quad j = 1,2, \ldots ,n$$

in which, $h\left( p \right) = h\left( {p_{1} ,p_{2} ,...,p_{n} } \right)$ is represented as a top event fuzzy fault function, $p_{j}$ denotes the fuzzy failure probability of the* j*-*th* bottom event. In this study, the triangular fuzzy function is used to represent the probability of failure of the bottom event, and the system fault tree logic gates are all "or gates", then the probabilistic importance of the bottom event is solved jointly by the Eqs. (26)–(28).

26 $$\begin{aligned} \partial h\left( p \right) & = 1 - \prod\limits_{i = 1}^{n} {\left( {1 - \mathop {F_{i} }\limits^{ \sim } } \right)} \\ & { = }\left[ {1 - \prod\limits_{i = 1}^{n} {\left[ {1 - \left( {m_{i} - \alpha_{i} } \right) - \alpha_{i} \lambda } \right]} ,1 - \prod\limits_{i = 1}^{n} {\left[ {1 - \left( {m_{i} + \beta_{i} } \right) + \beta_{i} \lambda } \right]} } \right] \\ \end{aligned}$$

27 $$\partial p_{i} = \left[ {\left( {m_{j} - \alpha_{j} } \right) + \alpha_{j} \lambda ,\left( {m_{j} + \beta_{j} } \right) - \beta_{j} \lambda } \right]$$

28 $$I_{h} \left( j \right) = \frac{\partial h\left( p \right)}{{\partial p_{j} }} = \prod\limits_{{\begin{array}{*{20}c} {i = 1} \\ {i \ne j} \\ \end{array} }}^{n} {\left\{ {\left[ {1 - \left( {m_{i} - \alpha_{i} } \right) - \alpha_{i} \lambda } \right],\left[ {1 - \left( {m_{i} + \beta_{i} } \right) + \beta_{i} \lambda } \right]} \right\}}$$

### FMEA-FFTA

**Figure 6:**
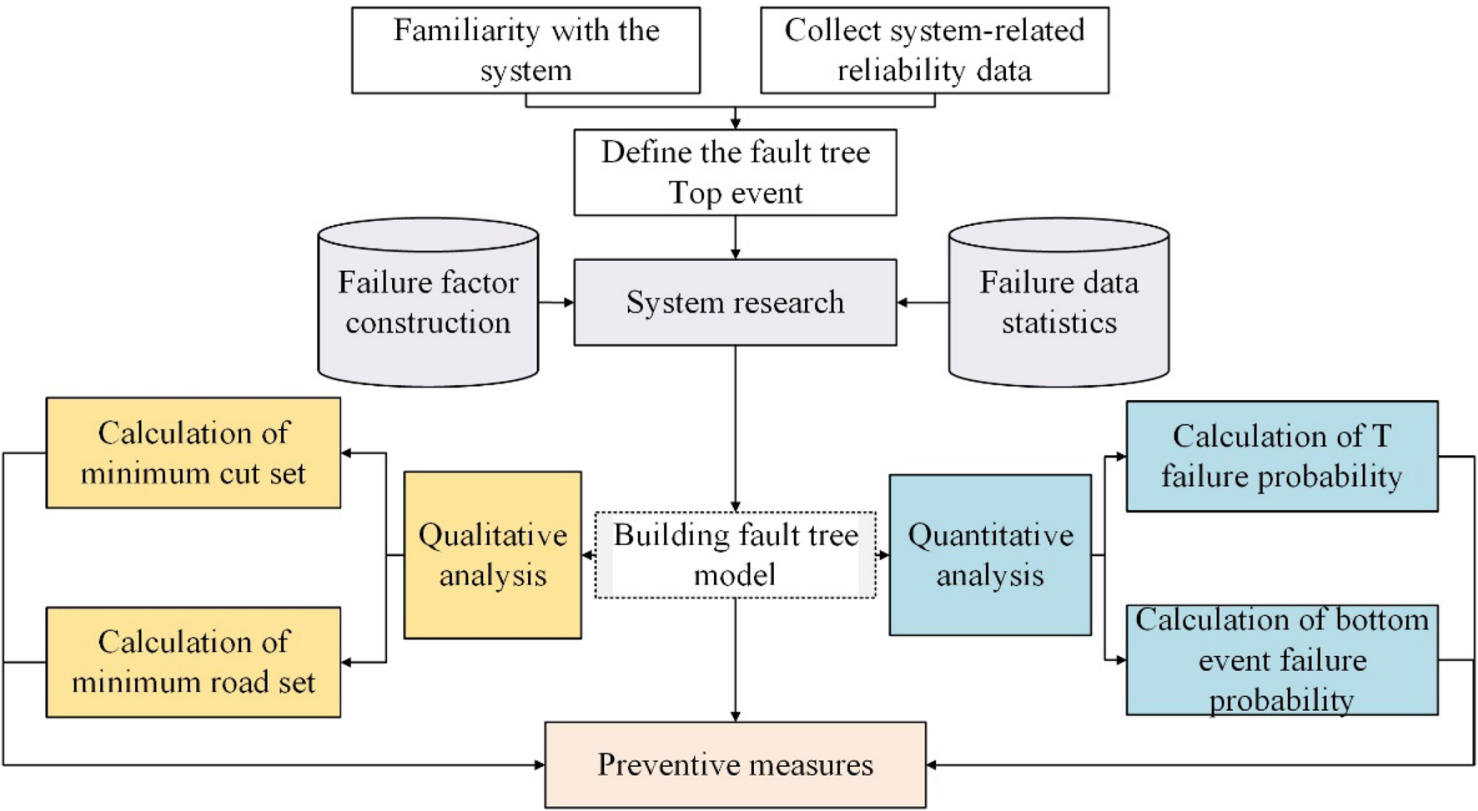
FMEA-FTA analysis flow chart.

In this study, a system reliability analysis method combined Failure Mode and Effects Analysis (FMEA) and Fuzzy Fault Tree Approach (FFTA) is introduced, the flow chart of FMEA-FTA is shown in Fig. 6.

## Reliability analysis of subsea control system

### FMEA analysis of subsea control system

**Figure 7:**
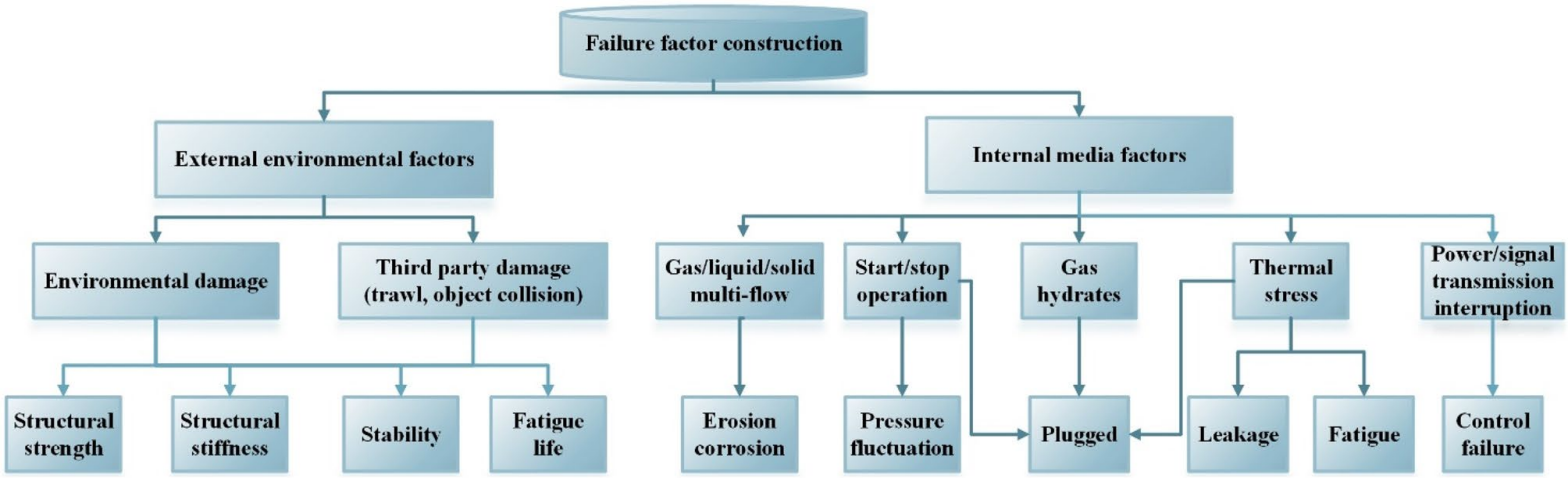
Failure factor construction of subsea production system.

Comprehensive consideration is given to the influence of various factors such as water depth, temperature, pressure, environment, technology, etc. Failure factors are constructed in terms of both external environmental factors and internal media factors. External influences from environmental damage, trawling, collision, third party personnel damage, causing failure of system equipment structural stiffness, strength and stability, etc. Internally affected by start-stop conditions, hydrates, power/signal transmission interruptions, multi-phase flow, etc., causing failure of equipment with blockage, leakage, fatigue, control, etc. Considering the multi-factor coupling effects of the external environment and internal media, the failure mode and failure cause analysis of the subsea control system is carried out to determine the factors affecting equipment safety, as shown in Fig. 7.

**Figure 8:**
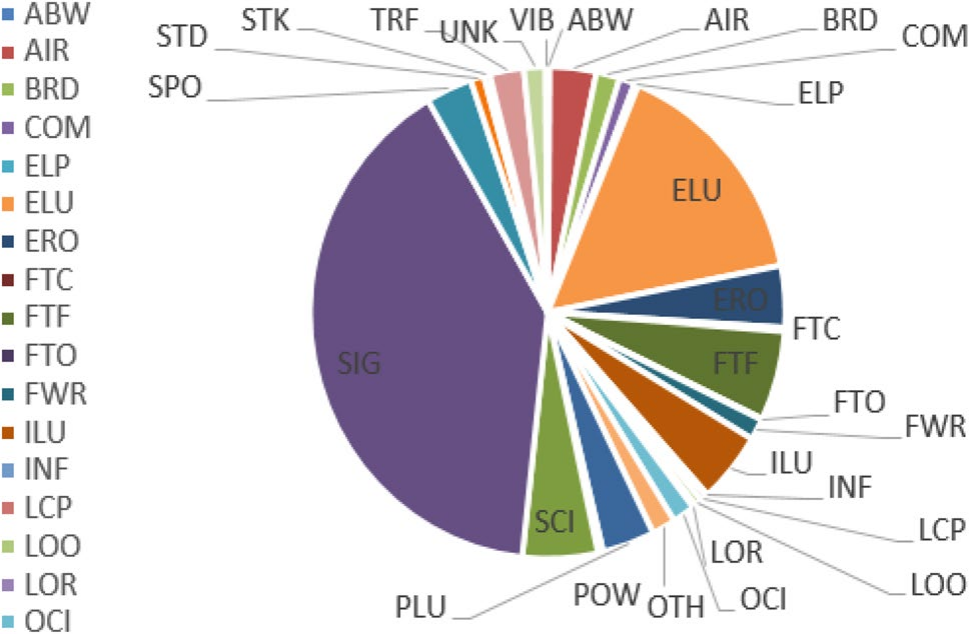
Failure statistics of subsea control system^60^.

The data in this study are mainly from the OREDA (2015) published by DNV^60^. These data have been collected through practice and provide an important reference value for the reliability and maintenance data unification of a large number of subsea oil and gas production systems in the North Sea, Gulf of Mexico and other areas. The potential failure modes of subsea control systems are shown in Table 5 and Fig. 8, and the FMEA of subsea control system is shown in Table 6.

**Table 5:** Failure statistics of control system^60^.

Common failure modes of subsea control system			
ABW	Abnormal wear	LOO	Low output
AIR	Abnormal instrument reading	LOR	Loss of redundancy
BRD	Breakdown	OCI	Open circuit
COM	Combined causes	OTH	Other failures
ELP	External leakage (Process medium)	PLU	Plugged
ELU	External leakage (Utility medium)	POW	Power shortage
ERO	Erratic output	SCI	Short circuit
FTC	Fail to close/lock on demand	SIG	Signal / Control failure
FTF	Fail to function on demand	SPO	Spurious operation
FTO	Fail to open/unlock on demand	STD	Structural failure
FWR	Failure while running	STK	Stuck
ILU	Internal leakage (Utility medium)	TRF	Transmission failure
INF	Insulation/isolation failure	UNK	Unknown expiry
LCP	Leakage of critical location	VIB	Vibration failure

**Table 6:** Subsea control system FMEA.

No	Failure mode	Causes of failure	Severity	Occurrence	Hazard level
1	ABW	Fatigue and vibration failure of subsea umbilical termination units	C3	F1	Low risk
2	AIR	Temperature and pressure sensor error signal/command/warning, indication failure, out of adjustment	C2	F3	Medium–low risk
		Flow sensor blockage, command failure, material failure			
		Umbilical terminal control failure, false signal/command/warning			
3	BRD	Broken umbilical terminal subunit, material failure	C2	F3	Medium–low risk
4	COM	Hydraulic coupling alignment failure	C2	F2	Low risk
		Hydraulic power unit error signal/command/warning, joint failure			
		Power/signal coupler hydraulically stuck, joint failure			
		Subsea umbilical termination unit control failure, electrical failure			
		Solenoid control valve power failure			
		Subsea power module error signal/command/warning, joint failure			
5	ELP	Subsea umbilical termination subunit leakage, mechanical failure	C3	F2	Medium–low risk
6	ERO	Pressure–temperature sensor error signal/command/warning, indication of failure	C2	F3	Medium–low risk
		Power supply unit power failure			
		Blocked pressure sensor, power failure, error signal/command/warning			
		Broken umbilical termination subunit, control failure, material failure, overheating			
		Position valve sensor control failure, incorrect energy/voltage			
7	FTC	Power/signal coupler leakage, hydraulic clamping	C3	F1	Low risk
8	FTF	Pressure/temperature sensor indication failure, material failure	C3	F3	Medium risk
		No signal/command/warning from sediment detection sensor			
		Solenoid control valves clogged, fouled, control failure, leaks hydraulic jamming, false signals/commands/warnings			
		Subsea power module control failure, power failure, incorrect voltage, incorrect signal/command/warning, command failure, leakage, open circuit, short circuit			
		Flow sensor indication failure, material failure			
9	FTO	Subsea umbilical termination subunit mechanical failure, leakage	C3	F1	Low risk
10	FWR	Subsea umbilical terminal control failure, earth fault, electrical failure, fatigue failure, incorrect energy/voltage, software failure	C3	F2	Medium–low risk
11	INF	Hydraulic/chemical line leaks	C3	F1	Low risk
		Clogged solenoid control valves, fouling, control failure, leaks			
		Subsea umbilical termination subunit leakage, mechanical failure			
12	LCP	Subsea umbilical termination leak	C2	F2	Low risk
13	LOO	Subsea umbilical termination subunits corrosion, fatigue failure, fouling	C2	F2	Low risk
14	LOR	Vibration failure of subsea umbilical termination subunits	C3	F1	Low risk
15	OCI	Coupling alignment failure, corrosion, incorrect grounding, looseness, open circuit	C3	F3	Medium risk
		Power/signal jumper trawl pulling influence			
		Subsea power module grounding error, open circuit			
		Subsea umbilical termination subunit loose grounding error, open circuit			
16	PLU	Clogged filters	C2	F3	Medium–low risk
		Plugged hydraulic/chemical line			
		Plugged solenoid control valves/position valves			
		Plugged subsea umbilical termination subunit			
17	POW	Subsea power module power failure	C3	F2	Medium–low risk
		Subsea umbilical termination unit power failure, no power/voltage			
18	PLU	Energy/signal coupler earth fault, no power/voltage, short circuit	C3	F3	Medium risk
		Energy/signal jumper earth/insulation fault, no power/voltage			
19	SIG	Pressure–temperature sensor control failure, electrical failure, instrumentation failure	C3	F5	High risk
		Flow sensor control failure, instrument failure, no signal, short circuit			
		Energy/signal line ground fault, no voltage, trawl pull			
		Sediment detection sensor control failure, electrical failure, instrumentation failure			
		Subsea power module control failure, power failure, false signal/command/warning, instrumentation failure, no signal/command/warning, defacement			
		Umbilical terminal control failure, instrumentation failure, loosening, open circuit, out of adjustment			
		Position valve sensor control failure, electrical failure			
20	SPO	Pressure–temperature sensor no signal/ warning, short circuit	C3	F3	Medium risk
		Plugged flow sensor			
		Pressure sensor error signal/command/warning, gauge failure			
		Subsea power module control failures, false signals, open circuit			
		Umbilical terminal control failure, electrical failure, software failure			
21	STD	Deformation of the base plate module, collision with falling objects	C3	F2	Medium–low risk
		Energy/signal jumper gap/alignment failure			
		Sheath anchored, deformed			
22	STK	Subsea solenoid control valves leakage, plugging	C3	F2	Medium–low risk
		Corrosion and fouling of subsea umbilical termination subunit			
23	TRF	Power supply unit power failure	C2	F3	Medium–low risk
		Plugged energy/signal coupler, gap/alignment failure, corrosion			
		Energy/signal jumper power failure, false signal, earth fault			
		Subsea power module power failure, error signals/warnings			
24	VIB	Subsea umbilical termination subunit fatigue, material failure	C2	F1	Low risk
25	ELU	Chemical injection coupling leakage	C3	F4	Medium–high risk
		Subsea substrate module leakage			
		Battery leakage			
		Umbilical termination subunit leakage			
		Hydraulic coupling breakage			
		Hydraulic power unit error signals/commands/warnings			
		Hydraulic/chemical jumper looseness, mechanical failure			
		Hydraulic/chemical line breakdowns, ruptures, leakage, trawl pulls			

A multi-factor failure mode analysis is carried out on the subsea control system according to the FMEA analysis steps, and the risk matrix method is used to comprehensively analyze the hazard degree of the identified failure modes, and the failure modes are classified into five levels: high risk, medium high risk, medium high risk, medium low risk and low risk. In FMEA, it can be concluded that SIG is classified as high risk level, ELU is classified as medium–high risk level, FTF as required, ILU, OCI, SCI and SPO are classified as medium risk level. The risk matrix method is used to identify the hazard level of the failure modes and the corresponding measures are taken for each failure mode to focus on prevention.

### FFTA of subsea control system

**Figure 9:**
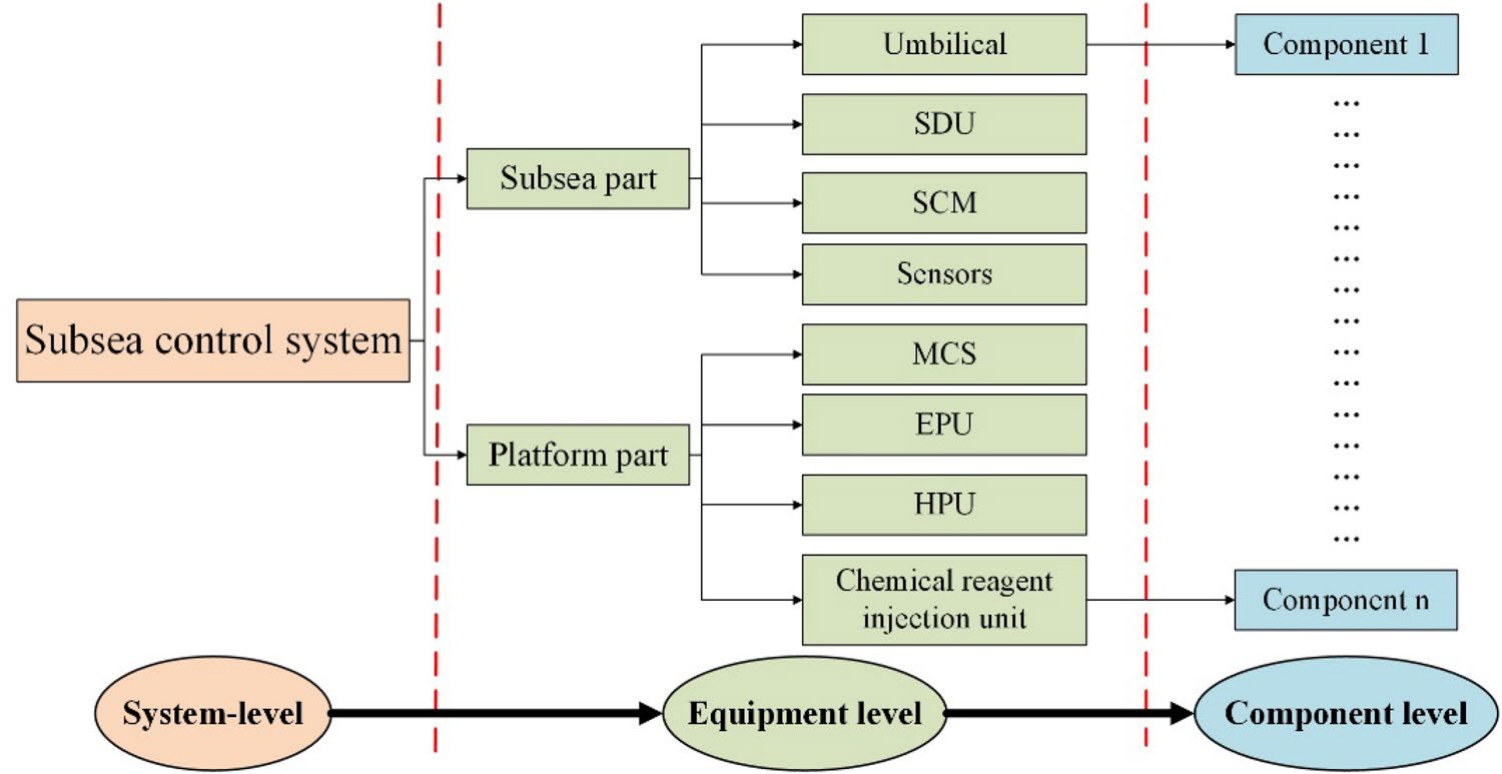
Stratification of subsea oil and gas production system.

In this study, the top event of the fault tree is selected as "failure of the subsea control system". Then, a hierarchical division of the subsea control system is agreed based on the "system level—subsystem level—device level—component level" model, as shown in Fig. 9.

**Figure 10:**
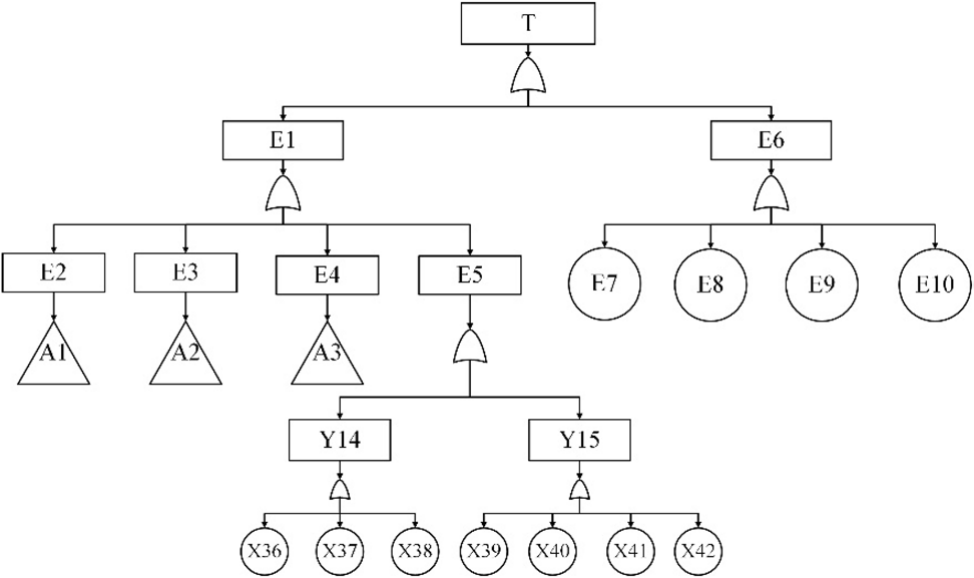
The fault tree branch of subsea control system.

**Figure 11:**
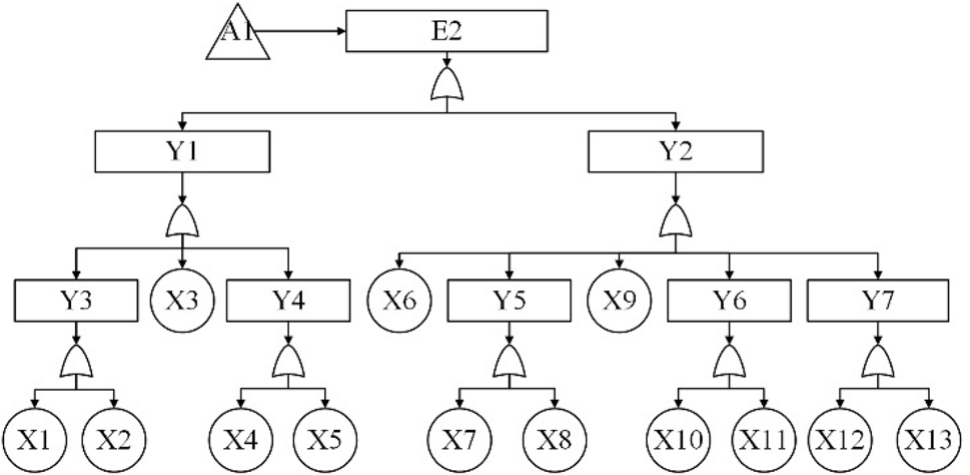
The fault tree branch of umbilical.

**Figure 12:**
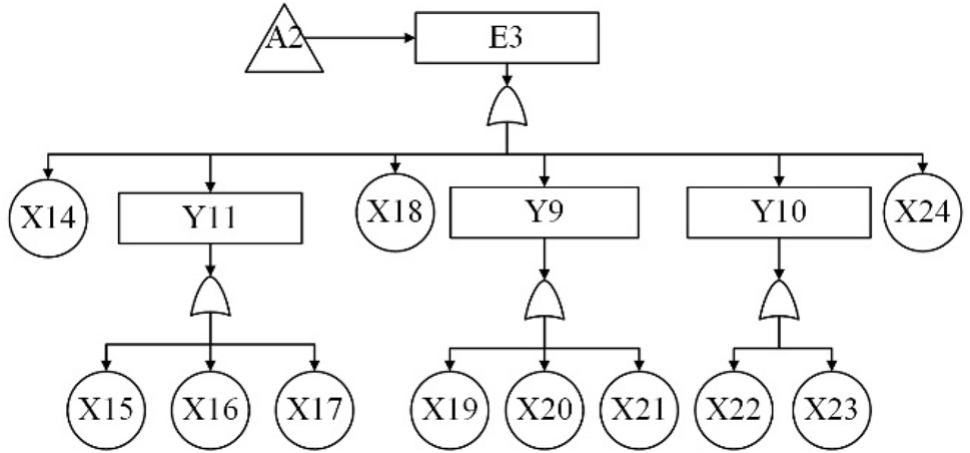
The fault tree branch of subsea distribution unit.

**Figure 13:**
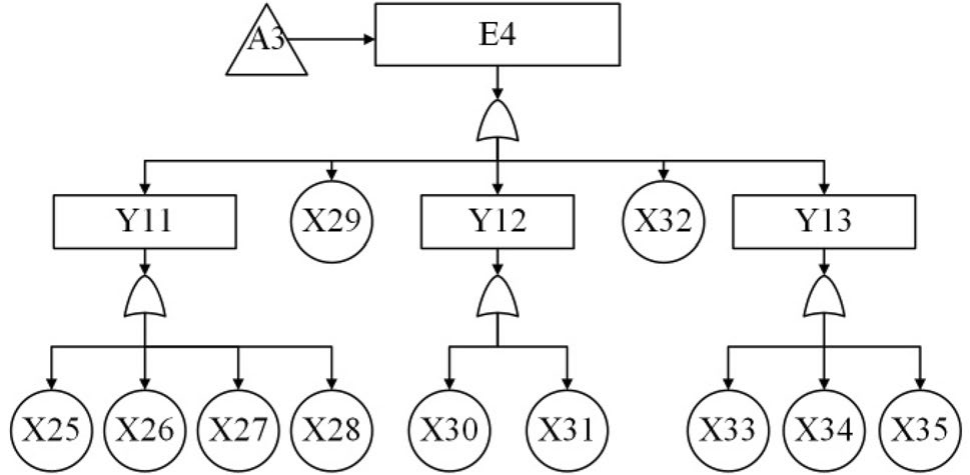
The fault tree branch of subsea control module.

**Figure 14:**
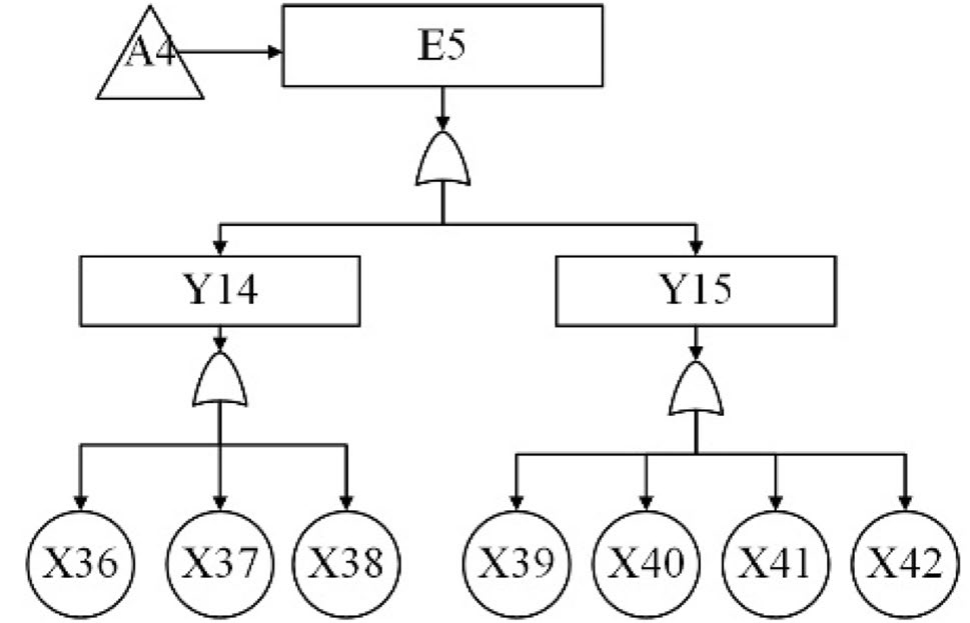
The fault tree branch of control system sensor.

According to the block diagram of the division structure above, system-level faults are identified as top event T, subsystem-level and equipment-level faults as intermediate events (represented by E and Y), and component-level faults as bottom event X. The system fault tree is then constructed from top to bottom, level by level. The event code table is shown in Table 7 and the fault tree model is shown in Fig. 10, 11, 12, 13 and 14.

**Table 7:** The event coding table.

Code	Event name	Code	Event name
T	Subsea control system failure	X9	Buoyancy device failure
E1	Subsea part of the control system failure	X10	Dynamic subsea umbilical termination failure
E2	Umbilical failure	X11	Dynamic overwater umbilical termination unit failure
E3	Subsea distribution unit failure	X12	Dynamic umbilical sheath/armor failure
E4	Subsea control module failure	X13	Stability & guidance equipment failure
E5	Control system sensor failure	X14	Subsea distribution module accumulator failure
E6	Topside part of the control system failure	X15	Distribution chemical injection coupling failure
E7	Chemical injection unit failure	X16	Distribution module fiber coupling failure
E8	Hydraulic power unit failure	X17	Distribution module hydraulic coupling failure
E9	Power unit failure	X18	Subsea distribution module power/signal line failure
E10	Main control station failure	X19	Fiber jumper failure
Y1	Static umbilical failure	X20	Hydraulic/chemical jumper failure
Y2	Dynamic umbilical failure	X21	Subsea distribution module power/signal jumper
Y3	Static umbilical part I failure	X22	Hose failure
Y4	Dynamic umbilical part I failure	X23	Hard tube failure
Y5	Static umbilical part II failure	X24	Subsea manifold failure
Y6	Dynamic umbilical part II failure	X25	Subsea control module chemical injection coupling failure
Y7	Dynamic umbilical protection structure failure	X26	Subsea control module fiber coupler
Y8	Subsea distribution unit coupling failure	X27	Subsea control module hydraulic coupling failure
Y9	Subsea distribution unit jumper failure	X28	Subsea control module jumper failure
Y10	Subsea distribution unit pipe failure	X29	Filter failure
Y11	Subsea control module coupling failure	X30	Solenoid control valve failure
Y12	Subsea control module valve failure	X31	Check valve failure
Y13	Subsea control module power unit failure	X32	Module baseplate failure
Y14	Temperature—pressure sensor failure	X33	Accumulator failure
Y15	Other sensors failure	X34	Power backup unit failure
X1	Static umbilical hydraulic/chemical line failure	X35	Subsea power module failure
X2	Power/signal line failure	X36	Temperature—pressure sensor failure
X3	Static umbilical sheath/ armor failure	X37	Pressure sensor failure
X4	Static subsea umbilical termination failure	X38	Temperature sensor failure
X5	Static overwater umbilical termination failure	X39	Flow sensor failure
X6	Bend restrictor failure	X40	Oil and gas leak sensor failure
X7	Dynamic umbilical hydraulic/chemical line failure	X41	Sand sensor failure
X8	Dynamic umbilical power/signal line failure	X42	Valve position sensor failure

The qualitative analysis of the subsea control system fault tree focuses on finding the minimum set of cuts and identifying all the basic events that lead to the occurrence of the top event. According to the fault tree rules, the minimum cut set of the fault tree of the subsea control system is found as shown in Eq. (29).

29 $${\text{T}} = \left\{ {{\text{X1}}} \right\},\left\{ {{\text{X}}2} \right\},\left\{ {{\text{X}}3} \right\},\left\{ {{\text{X}}4} \right\},\left\{ {{\text{X5}}} \right\},\left\{ {{\text{X6}}} \right\},\left\{ {{\text{X7}}} \right\}, \cdot \cdot \cdot ,\left\{ {{\text{X41}}} \right\},\left\{ {{\text{X42}}} \right\}$$

The failure data is mainly obtained from the latest version of the OREDA manual and combined with the actual field research situation to conduct failure data statistics to obtain the bottom event failure mean value $m$. Based on the traditional fault tree, the probability of failure of each bottom event is defined by introducing triangular fuzzy set theory, and the bottom event failure rate is described by the fuzzy subset. Assuming that the triangular fuzzy function $F_{i}$ is mutually symmetric and the affiliation of point with − 50% difference from the failure mean $m_{i}$ is 0.3 and the affiliation of point with + 50% difference from the failure mean $m_{i}$ is 0.2, as shown in Eq. (30).

30 $$\, \left\{ \begin{gathered} {1} - \left( {m_{i} - x} \right)/\alpha_{i} { = }\alpha_{i} /\left( {\alpha_{i} - {0}{\text{.5}}m_{i} } \right){ = 0}{\text{.3}} \hfill \\ {1} - \left( {x - m_{i} } \right)/\beta_{i} { = }\beta_{i} /\left( {\beta_{i} { + 0}{\text{.5}}m_{i} } \right){ = 0}{\text{.2}} \hfill \\ \end{gathered} \right.$$

By solving the above equation, it can be obtained that $\alpha_{i} { = 0}{\text{.714}}m_{i} ,\beta_{i} { = 0}{\text{.625}}m_{i}$, and according to this relation the upper and lower values of the probability of failure of the basic event can be solved, as shown in Table 8.

**Table 8:** The probability of bottom event^60^.

Event	$m$(10^–6^/h)	*α* (10^–6^/h)	*β* (10^–6^/h)	Event	$m$ (10^–6^/h)	*α* (10^–6^/h)	*β* (10^–6^/h)
X1	0.97	0.6926	0.6063	X24	2.74	1.9564	1.7125
X2	0.59	0.4213	0.3688	X25	0.28	0.1999	0.1750
X3	0.35	0.2499	0.2188	X26	23.78	16.9789	14.8625
X4	0.55	0.3927	0.3438	X27	0.17	0.1214	0.1063
X5	0.39	0.2785	0.2438	X28	0.13	0.0928	0.0813
X6	0.47	0.3356	0.2938	X29	0.02	0.0143	0.0125
X7	0.32	0.2285	0.2001	X30	0.53	0.3784	0.3313
X8	0.35	0.2499	0.2188	X31	0.53	0.3784	0.3313
X9	0.62	0.4427	0.3875	X32	0.29	0.2071	0.1813
X10	6.41	4.5767	4.0063	X33	0.09	0.0643	0.0563
X11	1.98	1.4137	1.2375	X34	0.03	0.0214	0.0188
X12	0.27	0.1928	0.1688	X35	9.90	7.0686	6.1875
X13	0.69	0.4927	0.4313	X36	3.88	2.7703	2.4251
X14	0.28	0.1999	0.1751	X37	0.72	0.5141	0.4501
X15	0.13	0.0928	0.0813	X38	0.68	0.4855	0.4251
X16	6.62	4.7267	4.1375	X39	3.51	2.5061	2.1938
X17	0.12	0.0857	0.0750	X40	0.36	0.2571	0.2251
X18	0.42	0.2999	0.2625	X41	56.4	40.2696	35.2501
X19	12.13	8.6608	7.5813	X42	2.80	1.9992	1.7500
X20	0.68	0.4855	0.4251	E7	19.95	14.2443	12.4688
X21	0.24	0.1714	0.1501	E8	83.45	59.5833	52.1563
X22	3.30	2.3562	2.0625	E9	17.68	12.6235	11.0501
X23	0.16	0.1142	0.1002	E10	123.9	88.4646	77.4375

In the fault tree analysis, the traditional logic gate operator is replaced by a fuzzy gate operator to obtain the individual bottom event fuzzy operators, as shown in Eq. (31).

31 $$\begin{aligned} & {\text{F}}_{1} = \left[ {\left( {0.9{7} \times 10^{{ - {6}}} - 0.{6926} \times 10^{ - 6} } \right) + 0.{6926} \times 10^{ - 6} \lambda ,\left( {0.9{7} \times 10^{ - 7} + 0.{6063} \times 10^{ - 6} } \right) - 0.{6063} \times 10^{ - 6} \lambda } \right] \\ & {\text{F}}_{2} = \left[ {\left( {0.{59} \times 10^{{ - {6}}} - 0.{4213} \times 10^{ - 6} } \right) + 0.{4213} \times 10^{ - 6} \lambda ,\left( {0.{59} \times 10^{{ - {6}}} + 0.{3688} \times 10^{ - 6} } \right) - 0.{3688} \times 10^{ - 6} \lambda } \right] \\ & \cdots \\ & {\text{F}}_{{{4}2}} = \left[ {\left( {2.80 \times 10^{{ - {6}}} - 1.9992 \times 10^{ - 6} } \right) + 1.9992 \times 10^{ - 6} \lambda ,\left( {2.80 \times 10^{{ - {6}}} + 1.7500 \times 10^{ - 6} } \right) - 1.7500 \times 10^{ - 6} \lambda } \right] \\ \end{aligned}$$

The failure probability interval on a truncated set of $\lambda$ is obtained by solving the equation programmatically according to the structure function, as shown in Eq. (32).

32 $${\text{F}} = \left[ {0.1121 \times 10^{{ - {3}}} + 0.{2783} \times 10^{ - 3} \lambda ,0.6334 \times 10^{ - 3} - 0.2431 \times 10^{ - 3} \lambda } \right]$$

When $\lambda = 1$, the bottom event failure rate is constant, and the probability of failure and reliability of ${\text{T}}$ is shown in Eqs. (33) and (34).

33 $${\text{F}}_{\lambda = 1} = {0}{\text{.0003904}}$$

34 $${\text{R}}_{{{\text{T}},\lambda = 1}} = 0.9996053$$

When $\lambda = 0$, the bottom event failure rate is a fuzzy interval value, and the probability of failure and reliability of ${\text{T}}$ is shown in Eqs. (35) and (36).

35 $${\text{F}} = \left[ {0.1121 \times 10^{{ - {3}}} ,\ 0.6334 \times 10^{ - 3} } \right]$$

36 $${\text{R}}_{{{\text{T}},\lambda = 0}} = \left[ {{0}{{.9993668, 0}}{.9998879}} \right]$$

**Figure 15:**
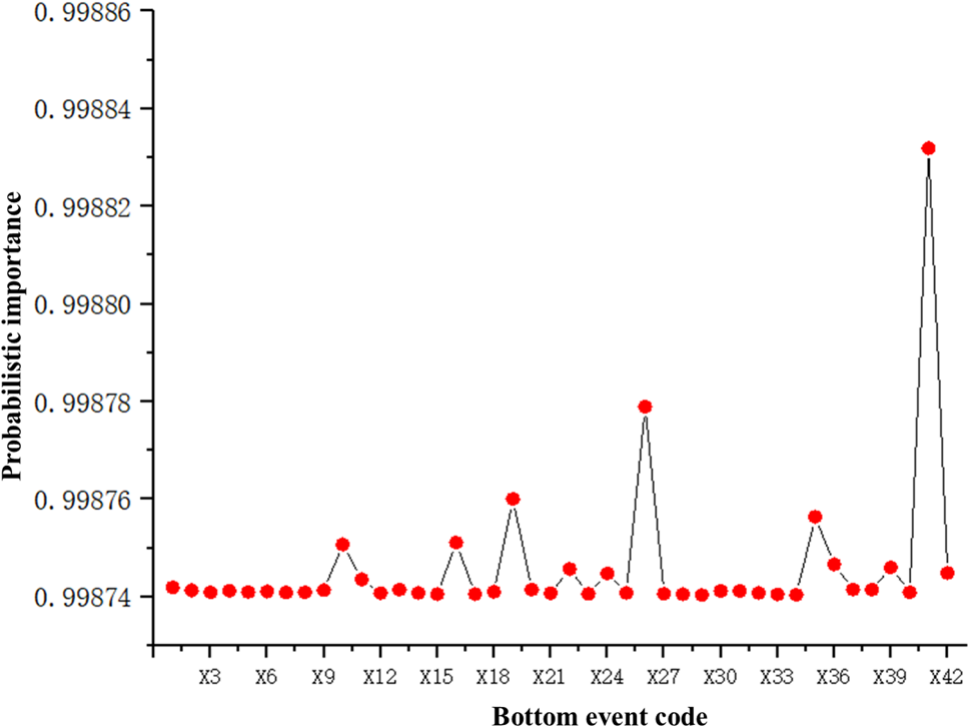
Bottom event probability importance.

MATLAB is used to perform algorithmic programming of the formula, taken as $\lambda { = }1$, to find the mean bottom event probability importance, and the results are imported into Origin for plotting, as shown in Fig. 15.

In Fig. 15, the values with higher probability of importance of the bottom event can be extracted and ranked by size: X41 > X26 > X19 > X35 > X16 > X10 > X36 > X39 > X22 > X42 > X24 > X11. To facilitate the identification of weak components in the system, the probability of importance of the components is sorted according to their probability of importance. It can be concluded that subsea distribution module fiber optic jumper, subsea distribution module fiber optic coupler, dynamic subsea umbilical cable terminal, temperature–pressure sensor, flow sensor, subsea distribution module hose, valve position sensor, subsea distribution module manifold, dynamic overwater umbilical cable terminal are relatively weak parts of the subsea control system. Measures should be taken to focus prevention and protection and regular testing to prevent and reduce production safety incidents and to promote orderly work in the water, oil and gas production system.

## Conclusion

In this study, a system reliability analysis method FMEA-FFTA is introduced, which combines Failure Mode and Effects Analysis (FMEA) with the Fuzzy Fault Tree Approach (FFTA). Firstly, the basic components and functions of the subsea control system are described, and a functional structure block diagram of the system is established. Then, the FMEA method is used to qualitatively analyze the reliability to identify the potential failure modes and causes of the subsea control system, and the risk matrix is applied to classify the failure modes into five levels: high risk, medium–high risk, medium risk, medium–low risk and low risk. A total of 25 main failure modes are identified, including high-risk modes such as signal/control failure, medium–high-risk modes such as external leakage (utility medium), and medium-risk modes such as fail to function on demand, internal leakage (utility medium), open circuit, short circuit, and spurious operation. Preventive and remedial measures are implemented based on the risk matrix results.

Subsequently, an agreed hierarchy of the subsea control system is divided, and the system fault tree model is built to find the minimum cut set of the fault tree. The fuzzy set theory is introduced to quantitatively analyze the subsea control system, calculate the system failure probability, and find out the relative weakness of the system. When the confidence level is set $\lambda { = }1$, the probability of system failure is ${\text{F}}_{\lambda = 1} = {0}{\text{.0003904}}$, and the reliability is ${\text{R}}_{{{\text{T}},\lambda = 1}} = 0.9996053$; when $\lambda { = }0$, the probability interval of system failure is ${\text{F}} = \left[ {0.1121 \times 10^{{ - {3}}} ,{\kern 1pt} {\kern 1pt} {\kern 1pt} {\kern 1pt} 0.6334 \times 10^{ - 3} } \right]$, and the reliability interval is ${\text{R}}_{{{\text{T}},\lambda = 0}} = \left[ {{0}{{.9993668, 0}}{.9998879}} \right]$. Finally, by solving the bottom event probability importance to find out the weak links in each subsystem, the analysis results show that the sand measurement sensor, subsea control module fiber optic coupler, subsea distribution module fiber optic jumper, subsea distribution module fiber optic coupler, dynamic subsea umbilical cable terminal, temperature–pressure sensor, flow sensor, subsea distribution module hose, valve position sensor, subsea distribution module manifold, failure of components such as dynamic above-water umbilical cable terminals are relatively weak points of the subsea control system and measures should be taken to focus on defense protection and regular detection.

Combining FMEA and FFTA offers a comprehensive approach to system reliability analysis. However, it is important to acknowledge the limitations of this combined method and consider potential future developments. For example, analyzing large-scale systems using FMEA-FFTA can be computationally demanding and time-consuming. As systems become more complex, the analysis may require significant computational resources and time, potentially limiting its practicality. The accuracy of the analysis heavily relies on the availability and quality of failure data for system components. Gathering comprehensive and reliable failure data can be challenging, particularly for novel or customized systems.

Future work will focus on further addressing its limitations and embracing future developments will contribute to its wider applicability and effectiveness in ensuring system integrity and performance. For example, integrating automation and AI techniques can reduce the manual effort required for the analysis, improve computation efficiency, and facilitate the identification of complex relationships and dependencies within the system. Developing reliable prediction models based on historical failure data can enhance the accuracy of the FMEA-FFTA analysis. These models can provide insights into failure patterns and improve the estimation of failure probabilities. Establishing standardized methodologies and guidelines for performing the FMEA-FFTA analysis can ensure consistency and enhance the adoption of this approach across industries.

## Data Availability

The datasets used during the current study available from the corresponding author on reasonable request.
